# The Effect of Aspirin on Bleeding and Transfusion in Contemporary Cardiac Surgery

**DOI:** 10.1371/journal.pone.0134670

**Published:** 2015-07-31

**Authors:** Jordan E. Goldhammer, Gregary D. Marhefka, Constantine Daskalakis, Mark W. Berguson, John E. Bowen, James T. Diehl, Jianzhong Sun

**Affiliations:** 1 Department of Anesthesiology, Sidney Kimmel Medical College, Thomas Jefferson University, Philadelphia, Pennsylvania, United States of America; 2 Division of Cardiology, Department of Medicine, Sidney Kimmel Medical College, Thomas Jefferson University, Philadelphia, Pennsylvania, United States of America; 3 Division of Biostatistics, Sidney Kimmel Medical College, Thomas Jefferson University, Philadelphia, Pennsylvania, United States of America; 4 Sidney Kimmel Medical College, Thomas Jefferson University, Philadelphia, Pennsylvania, United States of America; 5 Division of Cardiothoracic Surgery, Department of Surgery, Sidney Kimmel Medical College, Thomas Jefferson University, Philadelphia, Pennsylvania, United States of America; Sapienza University of Rome, ITALY

## Abstract

**Objective:**

Despite evidence that preoperative aspirin improves outcomes in cardiac surgery, recommendations for aspirin use are inconsistent due to aspirin’s anti-platelet effect and concern for bleeding. The purpose of this study was to investigate preoperative aspirin use and its effect on bleeding and transfusion in cardiac surgery.

**Methods:**

This retrospective study involved consecutive patients (n=1571) who underwent CABG, valve, or combined CABG and valve surgery at a single center between March 2007 and July 2012. Of all patients, 728 met the inclusion criteria and were divided into two groups: those using (n=603) or not using (n=125) aspirin within 5 days of surgery. Data were collected on chest tube drainage, re-operation for bleeding, and transfusion of red blood cells (RBCs), fresh frozen plasma (FFP), and platelets.

**Results:**

No significant difference was observed between the two groups in chest tube drainage or re-operation for bleeding. An increase in patients transfused with RBCs was observed in the aspirin group (61.9 vs 51.2%, adjusted OR 1.77, p=0.027); however, among those transfused RBCs, no significant difference in mean units transfused or massive transfusion was observed. No significant difference was seen in transfusion requirement of FFP or platelets.

**Conclusions:**

In patients undergoing CABG, valve, or combined CABG/valve surgery, preoperative aspirin, within 5 days of surgery, was associated with an increased probability of receiving an RBC transfusion. Preoperative aspirin was not associated with an increase in chest tube drainage, re-operation for bleeding complications, or transfusion of FFP or platelets.

## Introduction

Aspirin is used universally as an analgesic, anti-pyretic, anti-inflammatory, and anti-platelet agent. The use of aspirin to prevent cardiovascular complications in patients at risk for acute coronary syndrome and cerebrovascular events is well documented. In coronary artery bypass grafting (CABG), early postoperative aspirin therapy is associated with superior in-hospital and long-term outcomes attributed to improved graft patency [1–4], and postoperative aspirin use, within 48 hours of CABG surgery, is now the standard of care. Additionally, there is a growing amount of clinical data to suggest that preoperative aspirin may improve postoperative outcomes in cardiac surgery patients. Several retrospective cohort studies have identified a significant decrease of in-hospital mortality when patients are treated with preoperative aspirin [5–7]. However, despite contemporary evidence that preoperative aspirin improves postoperative outcome in cardiac surgery, the recommendations for preoperative aspirin use are inconsistent [8–10].

The overwhelming concern regarding perioperative aspirin is increased bleeding and its associated complications. A series of five randomized controlled trials from 1988 through 1994 showed an increase in transfusion, re-exploration, and chest tube drainage in patients treated with preoperative aspirin [11–15]. Observational studies have published both positive and negative associations between aspirin, transfusion, and bleeding [4–6, 16, 17]; however, it is difficult to draw conclusions from the existing body of literature due to the lack of contemporary studies utilizing blood conservation techniques. Thus, this study was designed to investigate transfusion and bleeding complications secondary to preoperative aspirin in a contemporary cardiac surgery population. We investigated bleeding and transfusion in patients undergoing CABG, valve, or combined CABG/valve surgery, and hypothesized that no significant increase in transfusion or bleeding complications would be observed in the aspirin treated population due to the use of contemporary blood conservation strategies including: cell salvage, anti-fibrinolytic medications, and conservative blood product transfusion.

## Materials and Methods

### Study Design

**Fig 1:**
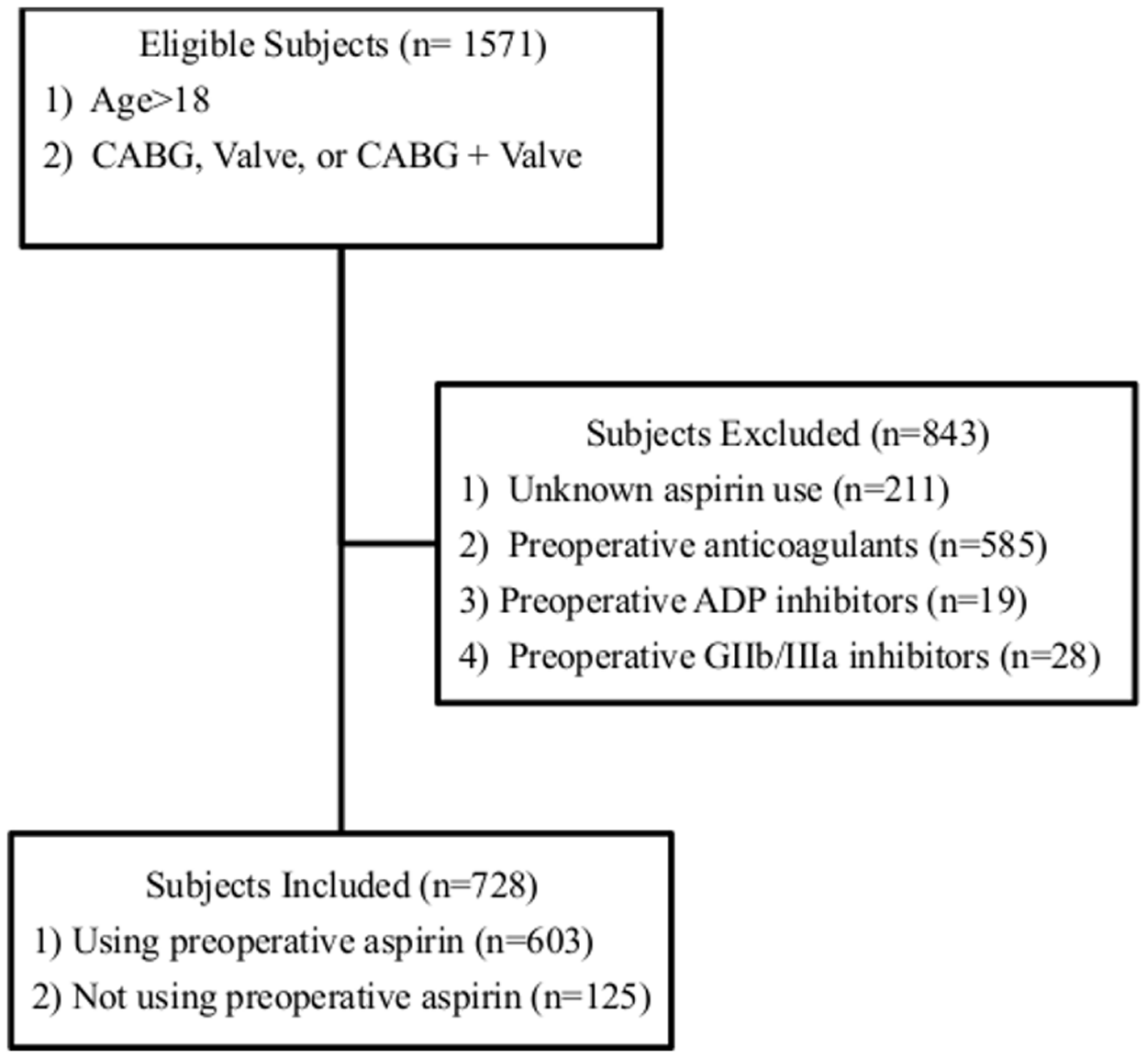
Study Inclusion/Exclusion Criteria.

This study was in compliance with the Declaration of Helsinki and reviewed and approved by the Thomas Jefferson University Institutional Review Board. Written informed consent was waived by the IRB and data was de-identified prior to analysis. This retrospective cohort study involved consecutive patients (n = 1571) having cardiac surgery including CABG, valve, or combined CABG and valve surgery at an academic medical center between March 2007 and July 2012. The patients excluded from the study cohort were those younger than 18, on preoperative anticoagulants, glycoprotein IIb/IIIa inhibitors, ADP inhibitors, or with incomplete documentation of aspirin use. Of all patients, 728 met the inclusion criteria and were divided into two groups; those using (n = 603) or not using (n = 125) preoperative aspirin (Fig 1). Preoperative aspirin use was defined as any dose of aspirin within five days of surgery.

### Data Collection

Data collected included demographics, medical history, and perioperative clinical data. Data were collected and organized to follow the template of the Society of Thoracic Surgeons database. Demographic and medical history data were collected upon hospital admission for cardiac surgery. Clinical data were collected in a prospective manner during the hospitalization for surgery.

Major outcomes of this study were bleeding and transfusion. Intraoperative transfusion data were collected from the electronic anesthesia record. Postoperative transfusion data were collected from the electronic medical record as indicated by physician order entry for transfusion of red blood cells (RBCs), fresh frozen plasma (FFP), and platelets. All transfusion data within five days of surgery were included in the analysis. Chest tube data was collected from the electronic medical record and followed until chest tube removal or postoperative day #5. Re-operation for bleeding complications was defined as any need for re-operation indicated by high chest tube output or cardiac tamponade. All operative notes from the hospital admission for cardiac surgery were reviewed to indicate if the patient underwent a re-operation due to bleeding complications.

### Statistical Analysis

Continuous variables were summarized with mean and standard deviation or geometric mean and interquartile range (25th to 75th percentile), depending upon their distribution. The main continuous outcomes (RBC, FFP, and platelet units; chest tube drainage volume and days) were log transformed prior to the analyses because of their skewed distribution. Univariable (unadjusted) analyses were based on t-tests for the continuous variables and chi-square tests (or Fisher’s exact tests, in the case of sparse data) for the categorical variables. Multivariable analyses were used to control for confounding factors between the aspirin and non-aspirin patient population. Demographic characteristics (sex, age, obesity), past medical history (diabetes, hypertension, cerebrovascular disease, peripheral vascular disease, chronic lung disease, renal failure, congestive heart disease, previous myocardial infarction, preoperative hematocrit), medications (beta blockers, angiotensin converting enzyme (ACE) or angiotensin receptor blocker (ARB) inhibitor, lipid lowering medications), and surgical characteristics (type of operation, perfusion time) were controlled for in the final models. Multivariable analyses were based on linear regression for the continuous outcomes and logistic regression for the dichotomous outcomes. The aspirin effects on the mean units transfused and chest tube drainage were expressed as geometric mean ratios (GMR), while the aspirin effects on the proportion of patients needing transfusion or re-exploration were expressed as odds ratios (OR). All reported *P*-values were 2-sided, and *P* < 0.05 was considered to be statistically significant. Statistical analysis was performed with SPSS 17.0 software for Windows.

## Results

### Clinical Characteristics

Of the 1571 patients in the database receiving CABG, valve, or combined CABG and valve surgery, 728 met the inclusion criteria and were divided into two groups: Those using (n = 603) or not using (n = 125) preoperative aspirin. Demographic and clinical data of the two patient groups are presented in Table 1. Patients treated with preoperative aspirin were more likely to have an increased age (66 vs 60 years, p = <0.001) and BMI (29.1 vs 27.6 kg/m2, p = 0.009). Additionally, aspirin users were more likely to have a past medical history of diabetes (35.0% vs 20.8%, p = 0.002), hypertension (89.6% vs 68.0%, p = <0.001), peripheral vascular disease (11.8% vs 4.8%, p = 0.021), previous myocardial infarction (26.9% vs 11.2%, p = <0.001), and a family history of coronary artery disease (53.6% vs 31.2%, p = <0.001). Patients on aspirin were more frequently treated with beta blockers (75.8% vs 42.4%, p = <0.001), ACE inhibitors or ARBs (46.4% vs 27.2%, p = <0.001), and lipid lowering medications (79.4% vs 36.8%, p = <0.001). Aspirin users and non-users did not differ in preoperative hematocrit (37.9% vs 38.1%, p = 0.606).

**Table 1:** Demographic and Clinical Characteristics.

Characteristics	Aspirin		P value
	Yes	No	
	N = 603	N = 125	
Age (yrs)	66±11	60±15	<0.001
Male gender	402(66.7)	76(60.8)	0.209
Body mass index (kg/m2)	29.1±5.9	27.6±6.2	0.009
Past medical history			
Diabetes	211(35.0)	26(20.8)	0.002
Hypertension	540(89.6)	85(68.0)	<0.001
Smoker	112(18.6)	21(16.8)	0.640
Cerebrovascular disease	86(14.3)	10(8.0)	0.060
Peripheral vascular disease	71(11.8)	6(4.8)	0.021
Chronic lung disease	120(19.9)	20(16.0)	0.314
Renal failure	29(4.8)	5(4.0)	0.696
Family history CAD	323(53.6)	39(31.2)	<0.001
Congestive heart failure	86(14.3)	19(15.2)	0.786
Previous MI	162(26.9)	14(11.2)	<0.001
Preoperative Hematocrit (%)	37.9±5.2	38.1±5.4	0.606
Medical therapy			
Beta-blockers	457(75.8)	53(42.4)	<0.001
ACE inhibitors or ARB	280(46.4)	34(27.2)	<0.001
Lipid lowering agent	479(79.4)	46(36.8)	<0.001
Type of Surgery			
CABG	372(61.7)	22(17.6)	
Valve Replacement	152(25.2)	87(69.6)	
CABG + Valve	79(13.1)	16(12.8)	<0.001
Operative Characteristics			
Previous cardiac surgery	38(6.3)	10(8.0)	0.486
ε-Aminocaproic acid (G)	9.4±3.9	9.4±3.1	0.956
Cell Saver (ml)	461±281	447±301	0.618
Perfusion time (min)	96±47	119±56	<0.001
Cross-clamp time (min)	76±29	91±39	<0.001

Values are n (%) for categorical variables and mean±SD for continuous variables.

### Procedure Characteristics

Of the 728 patients in the study population, 54.1% underwent CABG surgery, 32.8% valve surgery, and 13.0% combined CABG and valve surgery. Patients on aspirin were more likely to have a CABG operation (p = <0.001). Both cross clamp time (76 vs 91 minutes, p = 0.001) and perfusion time (96 vs 119 minutes, p = 0.001) were decreased in patients on preoperative aspirin. The incidence of previous cardiac surgery was similar between the two groups (6.3% vs 8.0%, p = 0.486), as was the administered dose of ε-aminocaproic acid (9.4 vs 9.4 G, p = 0.956) and the amount of cell salvage transfused (461 vs 447 ml, p = 0.618).

### Transfusion Outcomes

Overall, 60.7% of patients required transfusion of RBCs, 28.3% FFP, and 27.6% platelets. Of the patients on aspirin therapy, 61.9% required transfusion of RBCs, 27.2% FFP, and 27.4% platelets. A significant increase in the number of patients who received a transfusion of RBCs was observed in the aspirin population (61.9% vs 55.2%; adjusted OR 1.77; 95% CI 1.07, 2.95; p = 0.027); however, no significant increase in mean RBC units transfused in those who required transfusion (2.9 vs 3.1 units, adjusted GMR 1.08, p = 0.494) was observed. As seen in Table 2, no significant difference was observed in the number of patients receiving transfusion or mean units transfused in those who required transfusion of FFP or platelets. No significant difference was observed in massive transfusion of RBCs (1–4 vs >4 units, adjusted OR 1.00, p = 0.993), FFP (1–4 vs >4 units, adjusted OR 1.72, p = 0.264), or platelets (1–10 vs >10 units, adjusted OR 1.14, p = 0.794).

**Table 2:** Transfusion Outcomes, Aspirin vs No Aspirin.

Blood Product	Aspirin		Unadjusted	P value	Adjusted	95% CI	P value
	Yes	No	OR^1^ or GMR^2^		OR^1^ or GMR^2^		
	N = 603	N = 125					
**Red Blood Cells**							
Patients Transfused	373(61.9)	69(55.2)	1.32^1^	0.165	1.77^1^	1.07, 2.95	0.027
Number of Units*							
1–4	283(75.9)	47(68.1)					
>4	90(24.1)	22(31.9)	0.68^1^	0.174	1.00^1^	0.49, 2.03	0.993
Mean Units*	2.9 [2,4]	3.1 [2,6]	0.94^2^	0.581	1.08^2^	0.87, 1.35	0.494
**Fresh Frozen Plasma**							
Patients Transfused	164(27.2)	42(33.6)	0.74^1^	0.148	1.21^1^	0.72, 2.04	0.461
Number of Units*							
1–4	108(65.9)	29(69.0)					
>4	56(34.1)	13(31.0)	1.16^1^	0.696	1.72^1^	0.66, 4.48	0.264
Mean Units*	3.9 [2,6]	4.0 [2,6]	0.99^2^	0.967	1.09^2^	0.80, 1.50	0.580
**Platelets**							
Patients Transfused	165(27.4)	36(28.8)	0.93^1^	0.744	1.19^1^	0.70, 2.03	0.529
Number of Units*							
1–10	115(69.7)	21(58.3)					
>10	50(30.3)	15(41.7)	0.61^1^	0.187	1.14^1^	0.42, 3.10	0.794
Mean Units*	10.5 [8, 15]	12.2 [10, 20]	0.86^2^	0.201	1.02^2^	0.77, 1.34	0.900

Values are n (%) or categorical variables and geometric mean, IQR [25, 75] for continuous variables.

P <0.05 considered significant.

* Among patients transfused.

^1^Odds Ratio (OR).

^2^Geometric Mean Ratio (GMR).

### Indicators of Bleeding

Other indicators of bleeding studied were chest tube drainage and re-exploration rate (Table 3). Patients taking preoperative aspirin had no increase in days of chest tube drainage (2.6 vs 2.6 days; adjusted GMR 1.03; p = 0.444) or volume of chest tube drainage (874 vs 712 ml; adjusted GMR 1.16, p = 0.065). Additionally, no difference was observed in re-operation for bleeding complications. Patients on aspirin required re-operation for bleeding at a rate of 4.1%, non-aspirin users had a re-operation incidence of 4.8% (p = 0.742).

**Table 3:** Postoperative Bleeding, Aspirin vs No Aspirin.

Bleeding Outcome	Aspirin		Unadjusted	P value	Adjusted	95% CI	P value
	Yes	No	OR^1^ or GMR^2^		OR^1^ or GMR^2^		
	N = 603	N = 125					
Re-exploration	25(4.1)	6(4.8)	0.86^1^	0.742			
Chest Tube Drainage							
Volume (ml)	874 [553,1311]	712 [417,1152]	1.23^2^	0.004	1.16^2^	0.99, 1.35	0.065
Days	2.6 [2,3]	2.6 [2,3]	1.02^2^	0.532	1.03^2^	0.95, 1.12	0.444

Values are n (%) for categorical variables and geometric mean, IQR [25, 75] for continuous variables.

P <0.05 considered significant.

^1^Odds Ratio (OR).

^2^Geometric Mean Ratio (GMR).

## Discussion

Recent studies have shown the benefit of preoperative aspirin at reducing cardiac, cerebral, and renal complications, as well as in-hospital mortality for cardiac surgery patients. Cao et al, investigating a diverse patient population undergoing CABG, valve, or combined CABG and valve surgery, demonstrated preoperative aspirin to be associated with a significant decline in 30-day mortality, renal failure, and adverse cardiocerebral events, including; stroke, coma, heart block, and perioperative myocardial ischemia [7]. However, current practice guidelines remain conservative in their endorsement of preoperative aspirin. The 2011 American College of Cardiology/American Heart Association Guidelines for Coronary Artery Bypass Surgery, citing recent evidence that preoperative aspirin decreases morbidity and mortality, offers a class I recommendation to administer aspirin prior to CABG surgery; however, no specific recommendations of dose or discontinuation time are offered [8]. Conversely, the Society of Thoracic Surgeons 2005 guidelines suggest discontinuing aspirin therapy 3–5 days before elective CABG in order to decrease transfusion related complications [9]. A 2012 update to these guidelines maintains a recommendation to discontinue aspirin before elective surgery and in those at high risk for bleeding [10]. Despite contemporary evidence for improved perioperative outcome, the major guidelines offer conflicting recommendations for preoperative aspirin.

The existing literature exploring bleeding and transfusion due to aspirin must be applied with caution to contemporary cardiac surgery for a number of reasons. First, it has been more than twenty years since the often cited, randomized, controlled trials of aspirin’s effect on bleeding and transfusion [11–15]. Over the last two decades the universal use of cell salvage methods and anti-fibrinolytic medications have helped to decrease blood loss and transfusion in cardiac surgery [18,19]. Additionally, many institutions now utilize restrictive transfusion due to increased awareness of infection, immunosuppression, and transfusion reaction secondary to blood product administration. For these reasons, an updated look at the bleeding complications of aspirin is warranted using both conservative transfusion strategies and modern cell salvage techniques.

The major transfusion related finding of this observational cohort study is that preoperative aspirin is associated with an increased incidence of blood product administration. Aspirin users and non-users had a similar baseline hematocrit; however, after adjustment for confounding factors, aspirin was associated with a 77% relative increase in the incidence of red blood cell transfusion. An increase in transfusion of fresh frozen plasma was detected; however, this minor trend did not reach statistical significance. Interestingly, we did not detect a significant increase in platelet transfusion. This may be related to the relative scarcity of point of care testing regarding qualitative platelet function, and the reliance on quantitative platelet assessment (i.e. platelet count), which remains largely unaffected by aspirin therapy. In contrast, intraoperative hemoglobin and hematocrit levels may be expeditiously obtained, interpreted, and acted upon; perhaps accounting for increased red blood cell transfusion in patients exhibiting bleeding and anemia secondary to platelet inhibition.

Complications of blood transfusion are rare, but can be life threatening. Most reported transfusion reactions occur secondary to administration of mismatched blood products, a complication that can be avoided with vigilance. The incidence of serious transfusion related complications occurs approximately 1/1,100 transfusions and may occur more frequently in a massive transfusion setting [20]. Aspirin exposed patients in our cohort displayed no increase in massive transfusion of RBCs, FFP, or platelets; potentially mitigating some transfusion related risk of mismatched product administration, volume overload, and infectious complications. Overall, the potential benefits of preoperative aspirin must be weighed against the associated clinical risk of blood product administration.

Surgical re-exploration is associated with significant postoperative mortality and prolonged hospital stay [21,22]. The overall re-exploration rate in this surgical cohort was 4.2%, well within the historical range of 2% to 6%. Preoperative aspirin was not associated with an increase in re-exploration or chest tube output within our study group. Given the rarity of re-operation and our relatively small sample size, we were unable to adjust the incidence of re-exploration using regression analysis; however, it can safely be concluded from our unadjusted data that there is not an overwhelming signal linking aspirin to immediate re-operation for bleeding.

Previous literature has shown that the beneficial effects of aspirin are not limited to CABG and are applicable to most cardiac surgery procedures [7, 23–25]; however, the current literature assessing transfusion and bleeding complications related to preoperative aspirin consist only of CABG patients. Our study is the first to include valve and combined CABG/valve procedures to assess the bleeding complications of aspirin. The major guidelines for valve surgery, The Guidelines on Management of Valvular Heart Disease, focus extensively on postoperative anticoagulation and anti-thrombotic treatment and, in fact, completely fail to address the use of aspirin in the preoperative period [26]. As the applications of preoperative aspirin continue to expand, subgroup analysis of this population is warranted to further investigate the bleeding risk and mortality effect of aspirin in patients undergoing isolated valve or combined CABG/valve surgery.

### Limitations

Limitations of this study are a direct result of its observational design. Aspirin users differed from non-aspirin users in demographics, medical history, and surgical characteristics. We have attempted to account for the differences between the groups with regression analysis; however, residual confounding cannot be excluded from the results. Randomized, controlled trials of preoperative aspirin or a larger population to allow for propensity matched, retrospective analysis would be helpful to eliminate underlying confounding variables.

This study was designed to detect major differences in bleeding, transfusion, and re-exploration between aspirin users and non-users. We observed an increased incidence of RBC transfusion and no significant increase in transfusion of FFP and platelets; however, it must be noted that we encountered large confidence intervals in our incidence of transfusion analysis. While we can safely conclude no large effect was observed in transfusion of FFP and platelets, it is possible aspirin has a more modest effect undetected in this study. Future studies, encompassing large patient cohorts, will help to define the transfusion related risk of aspirin.

Our clinicians practice restrictive transfusion medicine and the decision to initiate blood product transfusion in our cohort was based upon clinical experience; however, a more accurate assessment of transfusion may have been possible with a lab based transfusion protocol. Overall, 60.7% of patients received an RBC transfusion within five days of surgery. While this transfusion rate seems elevated, it should be noted that our incidence of transfusion analysis includes blood products administered through postoperative day five, a longer transfusion interval than previously queried. Historical studies, utilizing restrictive transfusion practices, have demonstrated an RBC transfusion incidence of 32% within 24 hours of surgery and up to 56% in the first three postoperative days, a transfusion rate which is consistent with our data [17,27].

An additional limitation of this study is a lack of information regarding aspirin dose. Aspirin use was recorded in a categorical (“yes” or “no”) manner. Precise aspirin dose information would have been beneficial in order investigate the effects of escalating dose on bleeding and transfusion.

### Conclusions

Our study adds to growing amount of observational data that preoperative aspirin is associated with increased incidence of RBC transfusion but not associated with increase in re-operation rate or bleeding complications in cardiac surgery [16]. Multiple recent studies suggest favorable outcomes in patients treated with aspirin throughout the perioperative period for cardiac surgery [6,7, 23–25]. The risk associated with increased transfusion must be balanced with the risk of ischemic complications from perioperative aspirin interruption. Our results confirm a predilection for increased incidence of RBC transfusion in aspirin treated patients; however, most importantly, no increase was observed in the side effects that confer the greatest morbidity—massive transfusion or re-operation for bleeding complications. Although major guidelines still recommend otherwise, the growing pool of observational data appears to favor aspirin use throughout the perioperative period for cardiac surgery, with an acceptable accompanied risk of increased RBC transfusion.
